# Effect of Resveratrol Dry Suspension on Immune Function of Piglets

**DOI:** 10.1155/2018/5952707

**Published:** 2018-02-01

**Authors:** Qiuting Fu, Qiankun Cui, Yi Yang, Xinghong Zhao, Xu Song, Guangxi Wang, Lu Bai, Shufan Chen, Ye Tian, Yuanfeng Zou, Lixia Li, Guizhou Yue, Renyong Jia, Zhongqiong Yin

**Affiliations:** ^1^Natural Medicine Research Center, College of Veterinary Medicine, Sichuan Agricultural University, Chengdu 611130, China; ^2^College of Science, Sichuan Agricultural University, Ya'an 625014, China; ^3^College of Veterinary Medicine, Sichuan Agricultural University, Chengdu 611130, China

## Abstract

Resveratrol, a polyphenolic plant antitoxin, has a wide range of pharmacological activities. In this study, we systematically evaluated the effects of resveratrol dry suspension (RDS) on immune function in piglets that were treated with different doses of RDS for 2 weeks. The results showed that the RDS has significant effects on the development, maturation, proliferation, and transformation of T lymphocytes. RDS could regulate humoral immune responses by upregulating the release of IFN-*γ* and downregulating the release of TNF-*α*. After piglets were vaccinated against classical swine fever virus and foot-and-mouth disease virus, the antibody titers were significantly increased. RDS treatment showed an excellent resistance to enhance T-SOD activity. Values of blood routine and blood biochemistry showed no toxicity. These results suggested that RDS could be considered as an adjuvant to enhance immune responses to vaccines, as well as dietary additives for animals to enhance humoral and cellular immunity.

## 1. Introduction

The immune system is a vital barrier against the invasion of microorganisms, and it assumes enormous importance in fight against diseases and malignant abnormal cells [1]. Modern medical research has brought natural products into people's vision to enhance or restore the immune system. It is shown that some phytochemicals are beneficial to the health of the body by promoting the immune function, reducing inflammation, and activating enzymes [2]. As a result, natural plants with pharmacological activities are recommended as dietary supplements or therapeutic agents to effectively care for the organism.

Resveratrol (trans-3,4,5-trihydroxystilbene), a natural polyphenolic compound extracted from* Polygonum cuspidatum*, was first found in red wine because of the beneficial effect on the heart [3]. It has been exposed to a variety of biological activities, including anticancer, antioxidative, anti-inflammatory, antimicrobial, and estrogenic activities [4]. By interacting with multiple molecular targets, resveratrol could regulate innate and adaptive immunity [5]. It has attracted increasing attention due to the rich biological activities and has been recognized for its benefits to human health and used as a healthcare product in some people's diet [6].

Resveratrol supplementation in rat diets showed an increase in IgM concentration and splenocyte proliferation and a decrease in the triglyceride level [7]. In chickens, resveratrol could promote growth and inhibit antigen-induced apoptosis [8]. In ducklings infected with virulent duck enteritis virus, resveratrol supplementation could increase the survival rate, relieve tissue lesions, and reduce viral load in blood [9].

Although the function of resveratrol to regulate the immune response has been demonstrated in various animal models, it has been rarely reported in piglets. Pigs can be used as animal models for human diseases because of the great similarity between pigs and humans in lipid metabolism, cardiovascular physiology [10], and digestive system [11]. In our previous research, resveratrol was prepared into a dry suspension with the presence of suitable excipients to solve the trouble of poor water solubility in our laboratory. Therefore, in this study, the piglets were given resveratrol dry suspension (RDS) and the immune-regulating function was determined for the purpose of development of a new additive for piglets.

## 2. Materials and Methods

### 2.1. Chemicals

The resveratrol dry suspension (RDS) was prepared in Natural Medicine Research Center of Sichuan Agricultural University (Chengdu, China), and the content of resveratrol was 3%. Resveratrol was purchased from Sigma Co., Ltd. (USA).* Echinacea purpurea* powder was purchased from Qilu Animal Health products Co., Ltd. (Jinan, China).

### 2.2. Animals

Animal experiments were conducted under the principles of proper laboratory animal care and were approved by the ethical committee of the Laboratory Animals Care and Use of Sichuan Agriculture University (Chengdu, China; license number SCXK (Sichuan) 2014-187). 40 crossbred weaned piglets (Duroc × Landrace × Big White) at 28 days of age were randomly divided into five groups of 8 animals each group (4 females and 4 males). The 5 groups were as follows: saline control group (Group I), low dose of RDS-treated group (0.1 g/kg/d; Group II), middle dose of RDS-treated group (0.33 g/kg/d; Group III), high dose of RDS-treated group (1.0 g/kg/d; Group IV), and* Echinacea purpurea*-treated group (0.05 g/kg/d; Group V), respectively. The RDS and* Echinacea purpurea* (positive control) were suspended in water and fed to animals at 9 a.m. every morning for 14 days. The standard diet of animals was formulated based on the NRC (2012) recommendation for the nutrient requirements of 7–11 kg pigs [12]. The piglets were bred at a stationary temperature of 20–25°C, a stable relative humidity of 50 ± 10%, and illumination of 12 h per day in accordance with the International Committee on Laboratory Animals. The animals were domesticated for 4 days before experiments. It is assured that all animals are treated humanely in the laboratory and that the fewest numbers of animals are used to achieve the desired objectives.

### 2.3. Growth Performance and Visceral Index Assay

During treatment period, piglets were weighed under limosis. The states of the animals were observed and recorded every day. The average daily feed intake (ADFI), average daily gain (ADG), and ratio of feed to gain (F : G) were measured.

Within 24 hours of the last administration, piglets were sacrificed and the organs were weighed, including heart, lung, liver, kidney, spleen, and inguinal lymph nodes. The indexes were calculated according to the following formula: index (mg/g) = (the weight of organ)/the body weight.

### 2.4. Vaccine Treatment and Detection of Serum Antibody Level

Each piglet was inoculated with classical swine fever vaccine (CSFV) in the first day of the trial reference to the recommended immunization program [13]. A week later, the piglets were inoculated with foot-and-mouth disease vaccine (FMDV) again. The delay of second vaccination time was to eliminate or mitigate the stress response of piglets to FMDV [14].

Blood samples from anterior vein were collected to determine the serum antibody level at 0 d, 7 d, and 14 d during the trial, respectively. The antibody levels of CSFV and FMDV in serum were analyzed by ELISA kits (Shenzhen finder Biotech Co., Ltd., China) in accordance with the manufacturer's instructions.

### 2.5. T Lymphocyte Subsets Assay

Within 24 hours of the last administration, 2 ml of blood sample of each piglet from anterior vein was collected and dealt with EDTA. The lymphocytes were separated by lymphocyte separation medium (Beijing Solarbio, China). Then, the cells were incubated with CD3e-FITC, CD4*α*-PRE, and CD8*α*-SPRD monoclonal antibodies (BD Biosciences, USA) at temperature 37°C for 0.5 h in the darkness, followed by centrifugation and resuspending in PBS. T lymphocyte subsets were analyzed by flow cytometry (BD Biosciences, USA).

### 2.6. Proliferative Activity of Peripheral Blood Lymphocyte and Spleen Lymphocytes

Within 24 hours of the last administration, blood sample of each piglet from anterior vein was collected with anticoagulation. Then 3 ml of blood sample was slowly injected into 6 ml of porcine peripheral blood lymphocyte separation solution (Beijing Solarbio, China) and centrifuged to obtain the intermediate white cell layer. The cells were washed and centrifuged by PBS three times and then suspended in RPMI-1640 medium (Beijing Solarbio, China) at the concentration of 2 × 10^6^ cells/L. Blastogenic response of lymphocytes to the mitogen of ConA (Beijing Solarbio, China) was assessed by CCK-8 (Dojindo Laboratories, Japan). Lymphocyte suspension was incubated with ConA (10 *μ*g/mL) in 150 *μ*L RPMI 1640 medium containing 10% fetal bovine serum (FBS, Gibco Company, USA) at 37°C with 5% CO_2_. After incubation for 48 h, 10 *μ*L CCK-8 was added to each well. After incubation for 2 h, the absorbance at 450 nm was measured by a microplate reader (Bio-Rad, USA).

Within 24 hours of the last administration, 3 piglets from each group were sacrificed and the spleen was isolated in a sterile environment. Spleen tissue with the weight of 5 g was disrupted, and spleen cell suspensions were passed through sterile nylon mesh. Red blood cells were lysed by Erythrocyte Lysate (Beijing Solarbio, China). The spleen cells were suspended in RPMI-1640 medium and the methods of culture and detection were identical to those described above.

### 2.7. Determination of Serum Immunoglobulin Levels

The blood of piglets was collected from the anterior vein at the end of the trial. The serum was isolated by centrifugation. The serum concentrations of IgG, IgA, and IgM were measured by ELISA kits (Shanghai MLBIO, China).

### 2.8. The Antioxidant Capacity of Serum

The serum total antioxidant capacity (T-AOC), malondialdehyde level (MDA), and superoxide dismutase (T-SOD) in serum were determined by ELISA kits (Nanjing Jiancheng Bioengineering Institute, China).

### 2.9. Determination of Serum Cytokine Levels

The serum cytokine levels of interleukin, interferon, and tumor necrosis factor were determined by ELISA kits (Shanghai MLBIO, China).

### 2.10. Hematologic Examination and Serum Biochemical Examination

The blood samples obtained at the end of the trial were collected into a precalibrated tube containing sodium citrate. The hematological parameters included white blood cell count (WBC), red blood cell count (RBC), hemoglobin concentration (HGB), hematocrit (HCT), mean corpuscular volume (MCV), mean corpuscular hemoglobin (MCH), MCH concentration (MCHC), platelet count (PLT), and leukocyte differential count (lymphocytes, neutrophils, and monocytes) [15].

Serum biochemical indicators were detected, including albumin (ALB), total protein (TP), alanine aminotransferase (ALT), aspartate aminotransferase (AST), alkaline phosphatase (ALP), urea nitrogen (BUN), creatinine (CRE), glucose (GLU), calcium (Ca), phosphorus (P), total bilirubin (TBIL), and total cholesterol (CHO).

## 3. Results

### 3.1. Growth Performance and Visceral Coefficients

The growth performance and visceral index of piglets were shown in Table 1. Animals were randomly grouped and showed no difference in initial body weight. While the animals gained weight during experiment, the average daily feed intake and average daily gain of all drug treatments did not significantly differ in comparison to the saline control group (*p* > 0.05). The RDS and* Echinacea purpurea* treatment had no effect on coefficients of organs when compared to the saline control group (*p* > 0.05).

### 3.2. Percentage and Ratio of T Lymphocyte Subsets

The percentage of T lymphocytes in the peripheral blood of piglets was shown in Table 2, as well as the percentage of CD3+CD4+ and CD3+CD8+ labeled T cells and the ratio of the two. The percentages of T lymphocyte, including CD3+, CD3+CD4+, and CD3+CD8+, and the ratio of CD3+CD4+/CD3+CD8+ did not show any difference (*p* > 0.05) among all the groups. In RDS treatment, these T lymphocyte subsets were slightly higher than positive control (*p* > 0.05).

### 3.3. Proliferative Activity of Peripheral Blood Lymphocyte and Spleen Lymphocytes

**Figure 1:**
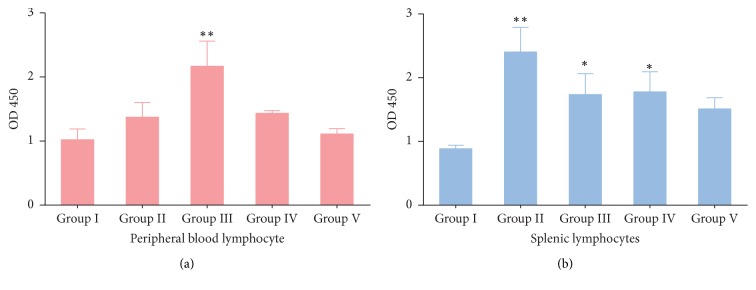
Proliferative activity of peripheral blood lymphocyte and spleen lymphocytes under the stimulation of ConA. (a) Proliferation of peripheral blood lymphocytes; (b) proliferation of splenic lymphocytes. Group I, saline control; Group II, RDS 0.1 g/kg treated group; Group III, RDS 0.33 g/kg treated group; Group IV, RDS 1.0 g/kg treated group; Group V,* Echinacea purpurea* powder 0.05 g/kg treated group. Data are represented as means ± SE; *n* = 6; comparison was made with the saline control group; one-way ANOVA followed by Duncan test. The symbols represent statistical significance at ^*∗*^ *p* < 0.05 and ^*∗∗*^ *p* < 0.01.

The proliferation of peripheral blood lymphocytes and splenic lymphocytes under the stimulation of ConA was shown in Figure 1. Compared with saline control group, RDS treatment (0.33 g/kg) significantly (*p* < 0.01) stimulated the proliferation of peripheral blood lymphocytes, while the other treatment groups did not show any differences. In splenic lymphocytes, all RDS treatments significantly increased (*p* < 0.05) lymphocyte proliferation, which showed RDS possessed potent effect on lymphocyte activity.

### 3.4. Antibody Levels in Serum

**Figure 2:**
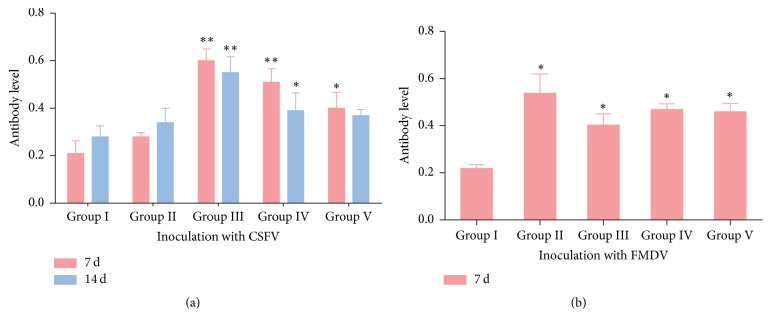
Antibody levels in serum. (a) The antibody level of CSFV; (b) the antibody level of FMDV. Group I. saline control; Group II, RDS 0.1 g/kg treated group; Group III, RDS 0.33 g/kg treated group; Group IV, RDS 1.0 g/kg treated group; Group V,* Echinacea purpurea* powder 0.05 g/kg treated group. RDS, resveratrol dry suspension; CSFV, classical swine fever vaccine; FMDV, foot-and-mouth disease vaccine. Data are represented as means ± SE; *n* = 6; comparison was made with the saline control group; one-way ANOVA followed by Duncan test. The symbols represent statistical significance at ^*∗*^ *p* < 0.05 and ^*∗∗*^ *p* < 0.01.

The detection of antibody levels in piglets was shown in Figure 2. The levels of CSFV antibody produced after 7 days of inoculation in piglets were significantly increased (*p* < 0.01) in RDS treatment (0.33 g/kg and 1.0 g/kg) compared to the saline control group, while the antibody level in* Echinacea purpurea* powder-treatment was also remarkably higher (*p* < 0.05) than that of saline control group. After 14 days of inoculation CSFV, only RDS treatment (0.33 g/kg and 1.0 g/kg) differed significantly in the saline control group (*p* < 0.01 or *p* < 0.05). Detection results after a week of vaccination with FMDV showed that all drug treatments significantly (*p* < 0.05) improved the antibody levels in piglets. These data demonstrated the positive effects of RDS on the secretion of antibodies.

### 3.5. Immunoglobulin Levels in Serum

**Figure 3:**
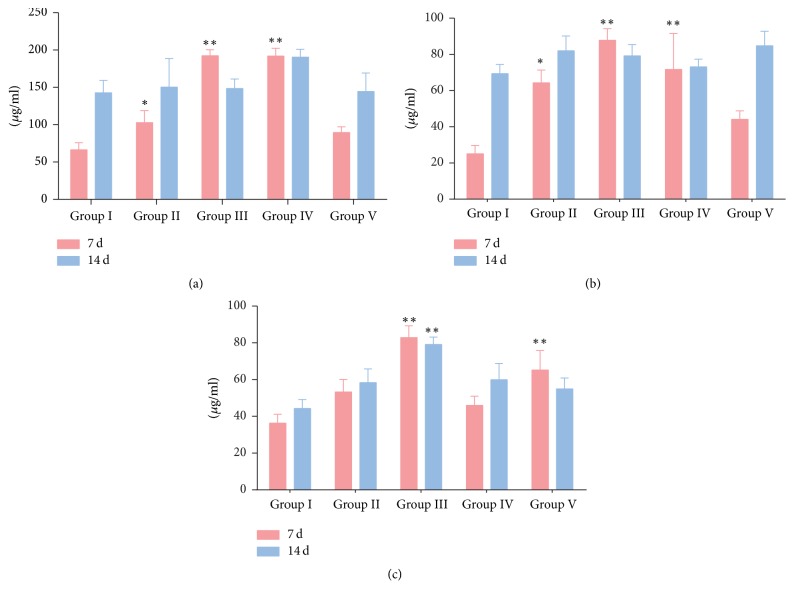
Immunoglobulin levels in serum. (a) Immunoglobulin G levels; (b) immunoglobulin M levels; (c) immunoglobulin A levels. Group I, saline control; Group II, RDS 0.1 g/kg treated group; Group III, RDS 0.33 g/kg treated group; Group IV, RDS 1.0 g/kg treated group; Group V,* Echinacea purpurea* powder 0.05 g/kg treated group. Data are represented as means ± SE; *n* = 6; comparison was made with the saline control group; one-way ANOVA followed by Duncan test. The symbols represent statistical significance at ^*∗*^ *p* < 0.05 and ^*∗∗*^ *p* < 0.01.

The immunoglobulins levels of serum in piglets were measured in the first and second weeks of the trial, respectively, and the results were shown in Figure 3. At 7 d of the trial, all RDS treatments significantly increased (*p* < 0.05 or *p* < 0.01) the levels of IgG and IgM in the serum, while the RDS treatment (0.33 g/kg) and* Echinacea purpurea* powder treatment significantly increased (*p* < 0.01) the content of IgA. At 14 d, the RDS treatment (0.33 g/kg) significantly promoted (*p* < 0.01) the secretion of IgA in serum, yet the other drug-treatment groups had no effect on the changes of immunoglobulin content compared with the saline control group.

### 3.6. Antioxidant Capacity of Serum

**Figure 4:**
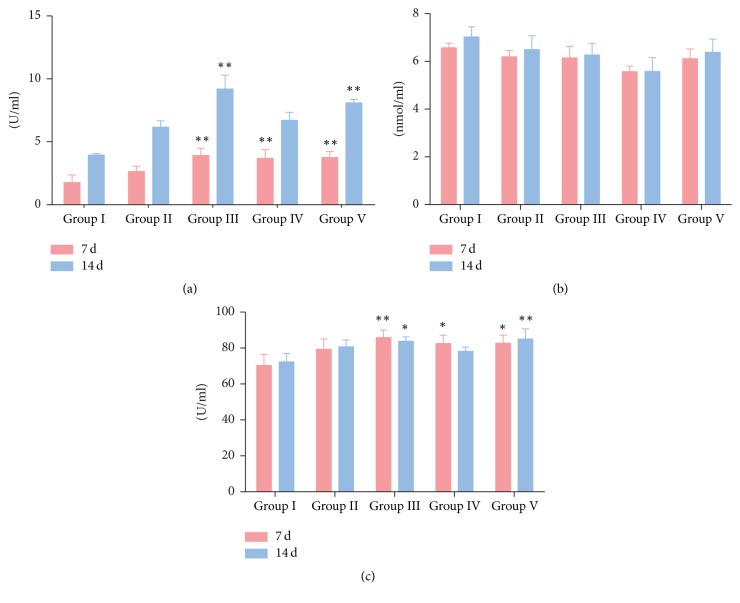
Serum total antioxidant capacity. (a) Serum T-AOC activity; (b) serum MDA activity; (c) serum T-SOD activity. Group I, saline control; Group II, RDS 0.1 g/kg treated group; Group III, RDS 0.33 g/kg treated group; Group IV, RDS 1.0 g/kg treated group; Group V,* Echinacea purpurea* powder 0.05 g/kg treated group. Data are represented as means ± SE; *n* = 6; comparison was made with the saline control group; one-way ANOVA followed by Duncan test. The symbols represent statistical significance at ^*∗*^ *p* < 0.05 and ^*∗∗*^ *p* < 0.01.

The result (Figure 4) showed that, at 7 d of the trial, RDS treatment (0.33 g/kg and 1.0 g/kg) and* Echinacea purpurea* powder treatment significantly improved (*p* < 0.01) the total antioxidant capacity of serum. Similarly, the RDS treatment (0.33 g/kg) and the* Echinacea purpurea* treatment significantly increased the total antioxidant capacity at 14 d, while the other groups were not significantly different compared with the saline control group. All the drug treatments had no effect on MDA production. RDS-treatment groups (0.33 g/kg and 1.0 g/kg) and positive control group significantly improved the activity of serum T-SOD after 7 d (*p* < 0.01 or *p* < 0.05), and only the RDS-treatment (0.33 g/kg) and positive control group significantly improved the activity of serum T-SOD after 14 d. The results confirmed that RDS had a good antioxidant capacity at the dose of 0.33 g/kg.

### 3.7. Cytokine Levels in Serum

**Figure 5:**
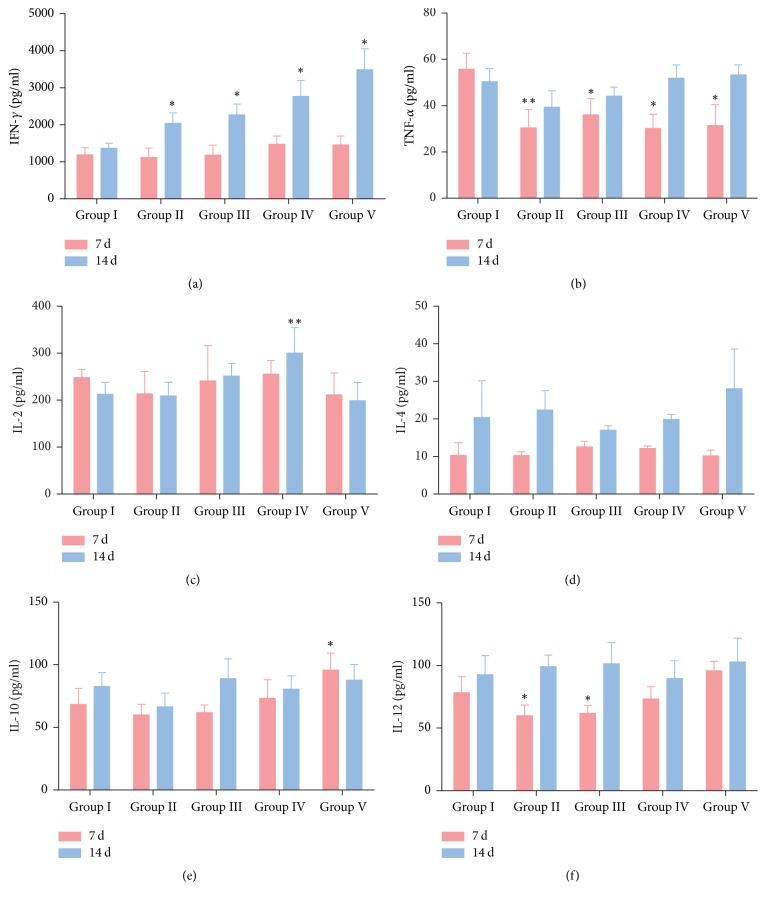
Cytokines levels in serum. (a) IFN-*γ* levels; (b) TNF-*α* levels; (c) IL-2 levels; (d) IL-4 levels; (e) IL-10 levels; (f) IL-12 levels; Group I, saline control; Group II, RDS 0.1 g/kg treated group; Group III, RDS 0.33 g/kg treated group; Group IV, RDS 1.0 g/kg treated group; Group V,* Echinacea purpurea* powder 0.05 g/kg treated group. Data are represented as means ± SE; *n* = 6; comparison was made with the saline control group; one-way ANOVA followed by Duncan test. The symbols represent statistical significance at ^*∗*^ *p* < 0.05 and ^*∗∗*^ *p* < 0.01.

The result (Figure 5) showed that all RDS treatments and* Echinacea purpurea* treatment reduced the release of TNF-*α* (*p* < 0.01 or *p* < 0.05) at 7 d, while the RDS treatment (0.1 g/kg and 0.33 g/kg) also reduced the release of IL-12 (*p* < 0.05). In the second week, all RDS-treatment and* Echinacea purpurea*-treatment groups increased the release of IFN-*γ* (*p* < 0.05) and the RDS treatment (1.0 g/kg) increased the release of IL-2 (*p* < 0.01).

### 3.8. Hematologic Examination and Serum Biochemical Examination

Tables 3 and 4 show the effects of RDS on blood and serum biochemical markers, respectively. RDS-treatment groups (0.33 g/kg and 1.0 g/kg) and positive control group significantly increased the number of white blood cells (WBC), neutrophils (NEUT), lymphocytes (LY), and monocytes (MONO). The creatinine (CRE) levels were significantly higher in the RDS medium and high dose groups than that of saline group (*p* < 0.05). The urea nitrogen (BUN) and triglyceride (TG) levels were increased in the RDS-treatment (0.1 g/kg) group (*p* < 0.01). Alanine aminotransferase (ALT) levels were increased in the median dose group; meanwhile blood sugar (GLU) levels were lower in the RDS-treatment (0.33 g/kg) group (*p* < 0.01).

## 4. Discussion

Our study systematically evaluated the effect of RDS on the immune function of piglets through various parameters. We found that RDS was the effective preparation of resveratrol and could significantly enhance immune function of piglets.* Echinacea purpurea* was shown to elicit an immune response by increasing the phagocytosis of granulocytes and the number of lymphocytes in fattening pigs as a feed additive [16]. Therefore, it was selected as a positive control drug to assess the effect on immune function of resveratrol. The results showed that RDS had a better immune-enhancing activity, suggesting that RDS had the potential to be used as an immunopotentiator.

In this study, RDS had no effect on the growth performance and organ coefficient of the piglets, which was similar to the previous study [17]. It was reported that standard diet supplemented with 300 or 600 mg resveratrol/kg significantly reduced the pig's liver coefficient being probable due to the decrease of the visceral adipose tissue weight [18].

CD3+CD4+ cell as a T helper/inducing cell secretes a variety of lymphokines which can regulate other cells involved in the immune response, while CD3+CD8+ cell as a cytotoxic T cell can secrete IFN-*γ* and kill the target cells carrying the antigen when it was activated [19]. The effect of resveratrol increasing the ratio of CD3+CD4+/CD3+CD8+ was confirmed in the obese model of C57BL/6 mice [20]. The reduction in CD3+CD4+/CD3+CD8+ ratio was usually associated with malignancies or the attack of the virus such as HIV infection [21], and the reduction also existed in the mouse model of systemic lupus erythematosus [22]. In our study, there was no significant difference between the normal and treated groups. When referring to the normal human range of 1.1–2 [23], the ratio of piglets was considered to have a normal fluctuation.

T lymphocytes can be transformed into lymphoblasts for cell division and proliferation in vitro culture under the stimulation of mitogen, such as concanavalin (ConA). Antigen stimulation changed from steady state of small lymphocytes into large lymphocytes, accompanied by increased cell volume and lighter nuclear staining, nucleolus, and cytoplasmic ribosome. Then, lymphocyte division and proliferation of effector cells took place [24]. Lymphocyte proliferation tests are often used to assess cellular immune function. It is reported that there was a trend for increased proliferation for cells treated with resveratrol [25]. Compared to the immunosuppressive mice, spleen lymphocyte proliferation was enhanced with resveratrol-treatment [26]. In our study, all RDS-treatment groups showed a positive effect on the activation and proliferation of T lymphocytes in spleen and in peripheral blood. Our study also demonstrated that RDS was effective in activating the function of T lymphocytes stimulated by antigens.

Natural products have been shown to serve as adjuvants that can enhance animal antibody levels under the stimulation of vaccines. Astragalus polysaccharide and oxymatrine have been reported to possess synergistical immunoenhancement in enhancing the immune efficacy of Newcastle disease vaccine [27]. The antibody titer against infectious bursal disease virus in broilers with treatment of* Echinacea purpurea* extract (0.1–1 g/kg) was significantly higher than that in control group [28]. Adding 0.5%* Echinacea* into diet had an enhancing effect on response of influenza vaccine [29]. Swine fever and swine foot-and-mouth disease are acute and infectious diseases which happened worldwide and brought huge losses to mankind [30]. In the present study, both RDS treatment (0.33 g/kg and 1.0 g/kg) and* Echinacea* treatment significantly improved the antibody titers against CSFV and FMDV, and the activity of RDS treatment was superior to* Echinacea* treatment. A recent study evaluated the effects of resveratrol on inflammatory response and antibody production against* Philasterides dicentrarchi* induced in turbot; the results showed a good regulatory effect of resveratrol on the inflammatory response the vaccine induced [31]. These results suggested that resveratrol could be considered as an adjuvant to enhance the immune response of vaccine in animals.

Immunoglobulins are formed in spleen and lymph nodes and secreted by mature plasma cells. They exist in the serum, body fluids, and tissues and can be directly involved in humoral immunity. Resveratrol supplementation remarkably promoted the production of immunoglobulin G in rats [32]. Similar studies also reported that dietary supplementation of 0.2% resveratrol improved the serum IgG levels in piglets [17]. In the first week of our trial, the levels of IgG, IgM, and IgA in serum were increased in varying degrees with different dose of RDS supplementation, while these effects could not be observed at the end of the second week. We speculate that this may be due to the improvement of the immune system in the growth process of piglets, and the impact of drug treatment on its immune response has diminished. These results suggested that RDS may be more effective in immunocompromised animals in regulating and participating in immune responses.

Recently, the antioxidant activity of resveratrol has been fully confirmed by various experiments. It has been shown that resveratrol can exhibit prooxidant properties, leading to oxidative breakage of cellular DNA in the presence of transition metal ions, such as copper, which hinted the anticancer and chemopreventive properties of resveratrol [33]. Resveratrol may protect against oxidant injury due to its capacity to inhibit COX-2-derived PGE 2 synthesis [34]. A study in rats showed that resveratrol significantly and dose-dependently decreased brain MDA level and increased brain SOD, catalase, and peroxidase activities [35]. RDS has been proven to enhance the activities of T-AOC and SOD in our experiment, while it did not affect the level of MDA in the serum. These studies showed that RDS enhanced the ability to scavenge oxygen free radicals and improved the total antioxidant capacity.

Resveratrol can regulate the secretion of cytokines by mediating and activating immune cells. It was reported that TNG-*α* levels in diabetic rats treated with resveratrol (5 g/kg) have decreased significantly [36], and this trend was also be demonstrated in our study. The mechanism may be due to the downregulation of JAK-STAT pathway and decreasing the levels of activated STAT1 in the nucleus [37]. Besides, resveratrol could reduce the release of proinflammatory cytokines on human periodontal ligament cells, such as IL-12 stimulated by LPS [38]. In our study, RDS was involved in the regulation of humoral immune responses by upregulating the release of IFN-*γ* and downregulating the release of TNF-*α*.

Blood routine and biochemical tests are often used to assist in the diagnosis of diseases and to observe the toxicity of drugs. In our study, the increase in WBC, NEUT, LY, and MONO suggested that a slight inflammation may have taken place in the RDS-treatment groups (0.33 g/kg and 1.0 g/kg) and* Echinacea purpurea*-treatment group. Resveratrol suppressed oxidative and inflammatory stress response to a high-fat, high-carbohydrate meal [39]. In the present study, the blood glucose (GLU) levels in the RDS-treatment (0.33 g/kg) group were also reduced, which was similar to the report. RDS had no significant effect on liver function, renal function, and electrolyte and other biochemical indexes in comparison with blank control. A small number of indicators (rise or fall) were still within the normal range of fluctuations, which can be accepted when referring to normal levels [40]. These tests suggested that RDS was lowly toxic or nontoxic to piglets.

## 5. Conclusion

In summary, RDS significantly affects the development, maturation, proliferation, and transformation of T lymphocytes and is involved in the regulation of humoral immune responses by upregulating the release of IFN-*γ* and downregulating the release of TNF-*α*. It significantly increased the antibody titers of the piglets under the stimulation of CSFV and FMDV when immunized against the vaccine. It showed an excellent resistance to oxidation and enhanced the T-SOD activity, and it has low toxicity. These positive effects hint that RDS could be considered as an adjuvant to enhance the body's immune response to vaccines, as well as dietary additives for animals to enhance humoral and cellular immunity and to play antioxidant and antiaging effects.

## Figures and Tables

**Table 1:** Growth performance and visceral index.

Items	Group I	Group II	Group III	Group IV	Group V
Initial body weight (kg)	6.52 ± 0.07	6.52 ± 0.18	6.75 ± 0.41	6.57 ± 0.38	6.61 ± 0.16
Final body weight (kg)	7.82 ± 0.37	7.65 ± 0.31	8.5 ± 0.4	8.13 ± 0.75	8.41 ± 0.13
Average daily feed intake (g)	242.86 ± 23.26	193.33 ± 12.03	248.07 ± 42.6	183.34 ± 33.35	128.09 ± 3.92
Average daily gain (g)	93.33 ± 29.65	80.95 ± 9.71	149.05 ± 27.64	111.9 ± 27.92	226.45 ± 26.95
Ratio of feed to gain	3.25 ± 1.04	2.42 ± 0.16	1.67 ± 0.03	1.68 ± 0.17	1.78 ± 0.24
Heart coefficient	5.37 ± 0.34	4.99 ± 0.24	5.45 ± 0.27	5.18 ± 0.11	5.51 ± 0.39
Lung coefficient	27.32 ± 2.52	26.5 ± 1.92	24.28 ± 2.05	24.47 ± 1.73	20.67 ± 1.25
Liver coefficient	27.32 ± 2.52	26.5 ± 1.92	24.28 ± 2.05	24.47 ± 1.73	20.67 ± 1.25
Kidney coefficient	5.94 ± 0.49	6.05 ± 0.18	6.3 ± 0.54	5.62 ± 0.22	6.11 ± 0.34
Spleen coefficient	1.94 ± 0.14	1.52 ± 0.18	1.68 ± 0.04	1.52 ± 0.16	2.01 ± 0.19
Lymph nodes coefficient	1.45 ± 0.13	1.84 ± 0.26	1.76 ± 0.22	1.44 ± 0.15	1.63 ± 0.16

Group I, saline control; Group II, RDS 0.1 g/kg treated group; Group III, RDS 0.33 g/kg treated group; Group IV, RDS 1.0 g/kg treated group; Group V, *Echinacea purpurea* powder 0.05 g/kg treated group. Data are represented as means ± SE; *n* = 6; comparison was made with the model group; one-way ANOVA followed by Duncan test.

**Table 2:** T lymphocyte subsets.

Items	Group I	Group II	Group III	Group IV	Group V
CD3+ (%)	65 ± 4.71	63.43 ± 5.02	71.17 ± 0.89	61.57 ± 4.87	62.1 ± 4.75
CD3+CD4+ (%)	27.97 ± 3.89	35.9 ± 5.71	43.3 ± 4.56	35.8 ± 3.39	29.37 ± 2.59
CD3+CD8+ (%)	23.67 ± 3.88	22.23 ± 2.63	24.23 ± 1.5	20.7 ± 2.01	18.1 ± 2.01
CD3+CD4+/CD3+CD8+	1.25 ± 0.29	1.6 ± 0.07	1.78 ± 0.09	1.74 ± 0.1	1.45 ± 0.17

Group I, saline control; Group II, RDS 0.1 g/kg treated group; Group III, RDS 0.33 g/kg treated group; Group IV, RDS 1.0 g/kg treated group; Group V, *Echinacea purpurea* powder 0.05 g/kg treated group. Data are represented as means ± SE; *n* = 6; comparison was made with the model group; one-way ANOVA followed by Duncan test.

**Table 3:** Blood routine examination.

Items		Group I	Group II	Group III	Group IV	Group V
WBC	(10^∧^9/L)	13.27 ± 0.71	13.04 ± 1.23	21.54 ± 3.29^*∗∗*^	21.09 ± 0.96^*∗∗*^	18.7 ± 1.18^*∗*^
NEUT	(10^∧^9/L)	5.02 ± 0.25	4.16 ± 0.77	11.2 ± 1.69^*∗∗*^	7.65 ± 0.11^*∗*^	7.8 ± 0.69^*∗*^
LY	(10^∧^9/L)	7.87 ± 0.55	8.43 ± 0.48	9.43 ± 1.61^*∗*^	13.21 ± 0.75^*∗∗*^	10.26 ± 0.51^*∗*^
MONO	(10^∧^9/L)	0.31 ± 0.04	0.27 ± 0.01	0.86 ± 0.15^*∗∗*^	0.63 ± 0.08^*∗*^	0.61 ± 0.09^*∗*^
HB	(g/L)	111 ± 1.32	113.67 ± 3.51	115.33 ± 6.64	110.67 ± 0.56	113.33 ± 3.19
PLT	(10^∧^9/L)	524.33 ± 55.85	448.67 ± 70.48	482.33 ± 52.38	430 ± 69.48	481.33 ± 52.59
RBC	(10^∧^12/L)	6.9 ± 0.08	6.95 ± 0.14	6.61 ± 0.64	7.01 ± 0.09	6.78 ± 0.36

Group I, saline control; Group II, RDS 0.1 g/kg treated group; Group III, RDS 0.33 g/kg treated group; Group IV, RDS 1.0 g/kg treated group; Group V, *Echinacea purpurea* powder 0.05 g/kg treated group. Data are represented as means ± SE; *n* = 6; comparison was made with the model group; one-way ANOVA followed by Duncan test. The symbols represent statistical significance at  ^*∗*^ *p* < 0.05 and  ^*∗∗*^ *p* < 0.01.

**Table 4:** Serum biochemical indexes.

Items		Group I	Group II	Group III	Group IV	Group V
TP	(g/L)	51.87 ± 0.27	53.3 ± 1.52	52.2 ± 2.2	49.43 ± 1.29	53.48 ± 0.79
ALB	(g/L)	36.07 ± 1.42	40.07 ± 2.24	36.43 ± 1.59	34.1 ± 1.12	38.72 ± 0.33
TBIL	(*μ*mol/L)	1.37 ± 0.13	1.57 ± 0.18	2.6 ± 0.52	1.57 ± 0.06	1.47 ± 0.12
ALT	(IU/L)	30.67 ± 2.05	26.2 ± 1.45	37.93 ± 6.76^*∗∗*^	29.7 ± 2.56	29.07 ± 1.03
AST	(I/L)	42.77 ± 1.38	50.23 ± 7.54	85.1 ± 10.79	59.1 ± 2.76	59.95 ± 2.48
ALP	(IU/L)	249.37 ± 11.59	250.67 ± 19.04	235.9 ± 30.28	265.13 ± 18.11	247.51 ± 5.78
*γ*-GT	(U/L)	47.47 ± 2.55	44.53 ± 1.7	49.9 ± 2.76	56.2 ± 2.72	54.62 ± 1.91
BUN	(mmol/L)	3.34 ± 0.45	4.73 ± 0.15^*∗∗*^	3.79 ± 0.44	3.67 ± 0.19	3.83 ± 0.14
CRE	(*μ*mol/L)	75.67 ± 3.94	76 ± 0.63	86.67 ± 5.42^*∗*^	85.67 ± 0.76^*∗*^	83.14 ± 2.43
GLU	(mmol/L)	5.44 ± 0.26	5.49 ± 0.19	4.69 ± 0.13^*∗∗*^	5.45 ± 0.11	5.34 ± 0.17
TC	(mmol/L)	1.66 ± 0.2	1.92 ± 0.03	1.9 ± 0.15	1.56 ± 0.02	1.95 ± 0.07
TG	(mmol/L)	0.36 ± 0.07	0.61 ± 0.09^*∗∗*^	0.48 ± 0.02	0.45 ± 0.02	0.48 ± 0.01
CK	(IU/L)	949 ± 330.45	816.33 ± 126.21	2493.33 ± 1061.58	1596 ± 360.01	743.33 ± 60.97
K	(mmol/L)	4.95 ± 0.18	4.79 ± 0.07	4.62 ± 0.4	5.27 ± 0.09	4.84 ± 0.15
Na	(mmol/L)	136.53 ± 2.21	132.1 ± 0.66	137 ± 2.18	136.47 ± 0.53	136.18 ± 1.21
Cl	(mmol/L)	96.7 ± 2.16	93.13 ± 1.48	98.33 ± 1.75	97.37 ± 0.14	98.47 ± 0.88
Ca	(mmol/L)	2.92 ± 0.07	3.07 ± 0.13	2.8 ± 0.12	2.83 ± 0.05	2.92 ± 0.1

Group I, saline control; Group II, RDS 0.1 g/kg treated group; Group III, RDS 0.33 g/kg treated group; Group IV, RDS 1.0 g/kg treated group; Group V, *Echinacea purpurea* powder 0.05 g/kg treated group. Data are represented as means ± SE; *n* = 6; comparison was made with the model group; one-way ANOVA followed by Duncan test. The symbols represent statistical significance at  ^*∗*^ *p* < 0.05 and  ^*∗∗*^ *p* < 0.01.
